# CVB3-Mediated Mitophagy Plays an Important Role in Viral Replication *via* Abrogation of Interferon Pathways

**DOI:** 10.3389/fcimb.2021.704494

**Published:** 2021-07-06

**Authors:** Soo-Jin Oh, Byung-Kwan Lim, Jeanho Yun, Ok Sarah Shin

**Affiliations:** ^1^BK21 Graduate Program, Department of Biomedical Sciences, College of Medicine, Korea University Guro Hospital, Seoul, South Korea; ^2^Department of Biomedical Science, Jungwon University, Goesan-gun, South Korea; ^3^Department of Translational Biomedical Sciences, Peripheral Neuropathy Research Center, College of Medicine, Dong-A University, Busan, South Korea

**Keywords:** Coxsackievirus B3 virus, mitochondrial dynamics, mitophagy, interferon, neural progenitor cells and stem cells

## Abstract

Coxsackievirus B3 (CVB3) is a common enterovirus that causes systemic inflammatory diseases, such as myocarditis, meningitis, and encephalitis. CVB3 has been demonstrated to subvert host cellular responses *via* autophagy to support viral replication in neural stem cells. Mitophagy, a specialized form of autophagy, contributes to mitochondrial quality control *via* degrading damaged mitochondria. Here, we show that CVB3 infection induces mitophagy in human neural progenitor cells, HeLa and H9C2 cardiomyocytes. In particular, CVB3 infection triggers mitochondrial fragmentation, loss of mitochondrial membrane potential, and Parkin/LC3 translocation to the mitochondria. Rapamycin or carbonyl cyanide m-chlorophenyl hydrazone (CCCP) treatment led to increased CVB3 RNA copy number in a dose-dependent manner, suggesting enhanced viral replication *via* autophagy/mitophagy activation, whereas knockdown of PTEN-induced putative kinase protein 1(PINK1) led to impaired mitophagy and subsequent reduction in viral replication. Furthermore, CCCP treatment inhibits the interaction between mitochondrial antiviral signaling protein (MAVS) and TANK-binding kinase 1(TBK1), thus contributing to the abrogation of type I and III interferon (IFN) production, suggesting that mitophagy is essential for the inhibition of interferon signaling. Our findings suggest that CVB3-mediated mitophagy suppresses IFN pathways by promoting fragmentation and subsequent sequestration of mitochondria by autophagosomes.

## Introduction

Coxsackievirus B3 (CVB3) infection contributes to diverse inflammatory diseases, such as myocarditis, meningitis, and encephalitis (Sin et al., 2015). Although CVB3 typically causes mild, self-limiting symptoms, severe CVB3 infection can lead to life-threatening diseases in both young and adult individuals, particularly in younger groups. Moreover, CVB3 infection in pregnancy is associated with higher risk of spontaneous abortion, fetal myocarditis, and neurodevelopmental defects in neonates (Lee et al., 2019; Wells and Coyne, 2019). In line with this, CVB3 has been shown to have tropism for neural progenitor cells (NPCs), as shown by our group and others (Feuer et al., 2003; Feuer et al., 2005; Feuer et al., 2009; Tsueng et al., 2011; Ruller et al., 2012; Oh et al., 2020).

Although CVB3 was thought to induce direct lysis of an infected cell, CVB3 may also escape from host cells *via* microvesicles including autophagy-related markers (Robinson et al., 2014). Autophagy is a highly conserved intracellular process that clears damaged organelles (Yorimitsu and Klionsky, 2005) and is implicated in numerous viral infections (Tian et al., 2019). Depending on the virus, autophagy can limit or promote viral infection. For CVB3, induction of autophagy facilitates its own replication, whereas blocking autophagy process *via* both *in vitro* (Wong et al., 2008; Delorme-Axford et al., 2014) and *in vivo* (Alirezaei et al., 2012) models greatly impairs viral replication.

Mitophagy is a selective form of autophagy that degrades damaged or dysfunctional mitochondria *via* delivering them to lysosomes for degradation (Youle and Narendra, 2011; Um and Yun, 2017). Mitophagy can be triggered by various stimuli, including viral infection, hypoxia, and alterations in mitochondrial dynamics. In this process, damaged mitochondria are enclosed by autophagic membranes to form mitophagosomes and delivered to the lysosomes for degradation. PINK1/Parkin-mediated pathway is the most studied and widely accepted for mitophagy process. In the PINK1/Parkin-mediated pathway, reduction in mitochondrial membrane potential can induce PINK1 stabilization and further facilitate the migration of Parkin to the mitochondria. In addition, there are other receptor-mediated pathways leading to mitophagy including BCL2 and adenovirus E1B 19kDa interacting protein 3 (BNIP3), Nip3-like protein X (NIX)/BNIP3L, FUN14 domain containing protein 1 (FUNDC1), and optineurin (OPTN). Multiple viruses have gained various strategies to counteract the antiviral mechanisms by controlling mitochondrial quality and content (Zhang et al., 2018; Ren et al., 2020). A recent study by Sin et al. showed that CVB3 promotes mitochondrial fragmentation and uses mitophagosomes to release extracellular microvesicles to promote viral dissemination (Sin et al., 2017).

Here, we investigated CVB3-induced alterations of mitochondrial dynamics in human neural progenitor cells, HeLa and H9C2 cardiomyocyte cells and observed increased viral replication *via* autophagy/mitophagy. Our data revealed that CVB3 triggers mitochondrial fission and mitochondrial translocation of Parkin. Furthermore, inhibition of mitophagy by silencing PINK1 results in the downregulation of CVB3 replication, whereas induction of mitophagy *via* CCCP treatment leads to impairment of interferon (IFN) activation and further contributes to enhanced CVB3 replication. We demonstrate that CVB3 may utilize mitophagy to evade innate immune responses.

## Materials and Methods

### Cells, Viruses, and Reagents

HeLa cells stably expressing Parkin (pcDNA3.1-Zeo-Flag-Parkin plasmid) were generated by transfection of pcDNA3.1-Zeo-Flag-Parkin and subsequent selection with zeocin (20 μg/ml) for 7 days (Ham et al., 2016). To generated HeLa cells in which PINK1 expression was knocked down, a lentiviral PINK1 shRNA construct (Sigma, TRCN 0000199193) was transfected into 293FT packaging cells. The resulting cell-free viral supernatant was used to infect HeLa cells. Following puromycin selection, resistant cells were pooled and used in the following experiments. HeLa-Parkin cells stably expressing the mitochondria-targeted fluorescent protein Keima (mt-Keima) was described in our previous study (Um et al., 2018). HeLa cells were cultured in Dulbecco’s modified Eagle’s medium (DMEM; Corning Mediatech, Corning, NY, USA) supplemented with 10% fetal bovine serum (FBS; Corning) and 1% penicillin/streptomycin. Human neural progenitor cells (hNPCs) has been described previously (Kim et al., 2018; Kim et al., 2019; Oh et al., 2020). Briefly, hNPCs were cultured in DMEM/F12 supplemented with 2% B27, 100 ng/mL fibroblast growth factor, 100 ng/mL epidermal growth factor, and 5 μg/mL heparin. THP-1 cells were cultured in RPMI1640 supplemented with 10% FBS, 2 mM glutamine, 10 mM HEPES, 1 mM sodium pyruvate, 0.05 mM 2-Mercaptoethanol, and 100 U/mL penicillin/streptomycin. Scrambled shRNA (shSCR) and dynamin-like protein 1 (Drp1)-specific shRNA-expressing (shDrp-1) THP-1 cells were previously described (Park et al., 2015) and kindly provided by Dr. Jewook Yu (Yonsei University School of Medicine, Korea). H9C2 cardiomyocytes, originally derived from embryonic rat heart tissues, were kindly provided by Dr. Hye Jin Yoo (Korea University Guro Hospital, Korea) and maintained in DMEM supplemented with 10% FBS and 1% penicillin/streptomycin.

CVB3 (H3 strain, Woodruff variant) (Knowlton et al., 1996) and GFP-CVB3 virus (Lim et al., 2005) were propagated at 37°C in HeLa cells. The virus titer was determined by plaque assay in HeLa cells as previously described (Oh et al., 2020).

### Drug Treatment

Carbonyl cyanide m-chlorophenyl hydrazone (CCCP), rapamycin, 3-methyladenine (3-MA), Mdivi-1, SBI-0206965 (ULK1 inhibitor), and dimethyl sulfoxide (DMSO) were purchased from Sigma-Aldrich (St. Louis, MO, USA). Cells were treated with various doses of the drugs prior to infection.

### Transmission Electron Microscopy

Mock-or CVB3-infected hNPCs were washed, pelleted and prepared for TEM analysis as described previously (Lee et al., 2020). After fixing the cells with PBS containing 2.5% glutaraldehyde and 2% paraformaldehyde for 30 min at 4°C, cells were post-fixed in 1% osmium tetroxide in PBS and differential concentrations of ethanol gradient (30%, 50%, 70%, 85%, and 95%) and 100% acetone were used to dehydrate the cells. The samples were polymerized with propylene oxide for 20 min and embedded in Epon mixture. 70 nm ultrathin sections were prepared and stained with uranyl acetate and lead citrate. Samples were observed using the Hitachi H-7600 electron microscope (Hitachi, Japan).

### Confocal Microscopy

For transfection, HeLa cells were seeded onto 12 mm coverslips in 24 well plates and transfected with various plasmids, followed by drug treatment or CVB3 infection at various time points. Expression vectors for mito-dsRED, TOM20-GFP, Parkin-GFP, and LC3-GFP have been previously described and were kindly provided by Dr. Woong Sun (Korea University School of Medicine, Seoul, Korea) (Cho et al., 2019). For the assessment of mitolysosome formation, cells were incubated in serum-free DMEM containing LysoTracker Deep Red (Thermo Fisher Scientific) and/or MitoTracker Green (Thermo Fisher Scientific) for 30 min. 24 hours post transfection, cells were washed with PBS, fixed with 4% paraformaldehyde, and permeabilized with 0.1% Triton X-100 as described previously (Seong et al., 2020a). CVB3 was detected using a mouse anti-Coxsackievirus primary antibody (1:1000 dilution; Merck), followed by an anti-mouse FITC- or Alexa 594-conjugated secondary antibody (Invitrogen). After washing, cells were stained with 4, 6-diamidino-2-phenylindole (DAPI) to visualize the nuclei and the images were examined using a confocal microscope (LSM700; Carl Zeiss, Oberkochen, Germany). Mitochondrial area were measured by Zeiss Zen software.

### Quantitative Realtime PCR

Total RNA was extracted utilizing DirectZol RNA kit (Zymo Research, Irvine, CA, USA) and cDNA was synthesized using the ImProm-II Reverse Transcription System (Promega, Madison, WI, USA) according to the manufacturer’s instructions. Power SYBR^®^ Green Master Mix (Invitrogen) was used to determine the relative expression of genes using following primers: *CVB3 Forward: CCC TGA ATG CGG CTA ATC C, CVB3 Reverse: AAA CAC GGA CAC CCA AAG TAG TC, Human IFN-β Forward: GCT TGG ATT CCT ACA AAG AAG CA, Human IFN-β Reverse: ATA GAT GGT CAA TGC GGC GTC, Human ISG15 Forward: GAG AGG CAG CGA ACT CAT CT, Human ISG15 Reverse: CTT CAG CTC TGA CAC CGA CA, Human PINK1 Forward: GGA CAC GAG ACG CTT GCA, Human PINK1 Reverse: TTA CCA ATG GAC TGC CCT ATC A, Human β-actin Forward: CAT GTA CGT TGC TAT CCA GGC, Human β-actin Reverse: CTC CTT AAT GTC ACG CAC GAT, Rat β-actin Forward: GCT GAC AGG ATG CAG AAG GAG, Rat β-actin Reverse: GAG CCA CCA ATC CAC ACA GAG.* Relative mRNA levels were determined using the comparative **ΔΔ**Ct method and normalized to *β-actin* mRNA levels.

### Mitochondrial Membrane Potential Measurements

Tetramethylrhodamine methyl ester (TMRM; Thermo Fisher Scientific) was used to measure MMP. Cells were plated in 96-well black plates and infected with CVB3 at different MOIs for 24 hours or treated with CCCP for 2 hours. Cells were incubated with 20 nM of TMRM at 37°C for 30 min before washing and mounting in Dulbecco’s phosphate-buffered saline (DPBS) for visualization. TMRM fluorescence was measured using the Varioskan LUX multimode microplate reader (Thermo Fisher Scientific).

### Mitochondrial Dynamics Profile

Using a Seahorse XFp extracellular analyzer (Seahorse BioSciences, Billerica, MA, USA), oxygen consumption rate (OCR) was measured as described previously (Seo et al., 2018). In details, hNPCs were plated at 8,000 cells/well and infected with mock or CVB3. On the day of OCR analysis, the medium was changed to basal DMEM media, and the cells were incubated at 37°C in a non-CO_2_ incubator for 1 h. After the assay, cells were counted and the rate of OCR was normalized to cell number.

### Immunoblot Analysis and Co-Immunoprecipitation

Immunoblot analysis and co-immunoprecipitation assays were performed as described previously (Oh and Shin, 2021). Cells were harvested and lysed with RIPA buffer (Sigma-Aldrich) containing protease and phosphatase inhibitors (Roche). Proteins were separated by SDS-PAGE, transferred onto polyvinylidene difluoride membranes, and blocked with 5% skim milk in TBS supplemented with 0.1% Tween-20 (TBS-Tw) for 1 h at room temperature. The membranes were then incubated with primary antibodies (Cell Signaling Technology) at 4°C overnight, followed by HRP-conjugated anti-rabbit or anti-mouse IgG secondary antibodies for 1 h at room temperature. Tubulin or β-actin (Abgent, CA, USA) antibody was used as a loading control. Densitometry was performed to quantify the protein bands using ImageJ software, and a representative image of three independent experiments is shown.

For co-immunoprecipitation, HEK293T cells were transfected with mitochondrial antiviral signaling protein (MAVS), TANK-binding kinase 1 (TBK1), or interferon regulatory transcription factor 3 (IRF3)-encoding plasmids using polyethylenimine (Sigma) in the presence or absence of CCCP. Cells were pelleted and resuspended in Pierce^®^ IP lysis buffer and supplemented with complete protease inhibitor cocktail (Roche).

### siRNA Transfection

HeLa cells were plated in 6 well plates. Control scrambled or *PINK1*-specific siRNAs were synthesized (Bioneer, Daejeon, Korea) and transfected into the cells using RNAiMAX transfection reagent (Invitrogen) according to the manufacturer’s protocol (Kim et al., 2017; Seong et al., 2017).

### Luciferase Reporter Assay

The luciferase reporter assay was performed as described previously (Seong et al., 2020b). Cells were transfected with plasmids carrying IFN-β, IFN-λ1, and IP-10 luciferase reporter gene in combination with an empty vector (EV) and retinoic acid-inducible gene-I (RIG-I)-specific plasmids. At 24 h post-transfection, cells were treated with DMSO or CCCP for 2 h, and cell lysates were assessed for luciferase activity using Dual-Glo luciferase reporter assay system (Promega).

### Measurement of Cytokine Secretion by ELISA

Cell-free culture supernatants were examined for IFN-λ1/3 concentrations using the ELISA kit (R&D Systems, Minneapolis, MN, USA) following the manufacturer’s instructions.

### Statistical Analysis

Data are presented as the mean ± standard deviation (SD) from at least two to three independent experiments. Statistical comparisons between different treatments were performed using the Mann-Whitney test or student’s t test, and results with p < 0.05 were considered statistically significant.

## Results

### CVB3 Infection Leads to Autophagosome Formation in hNPCs

**Figure 1:**
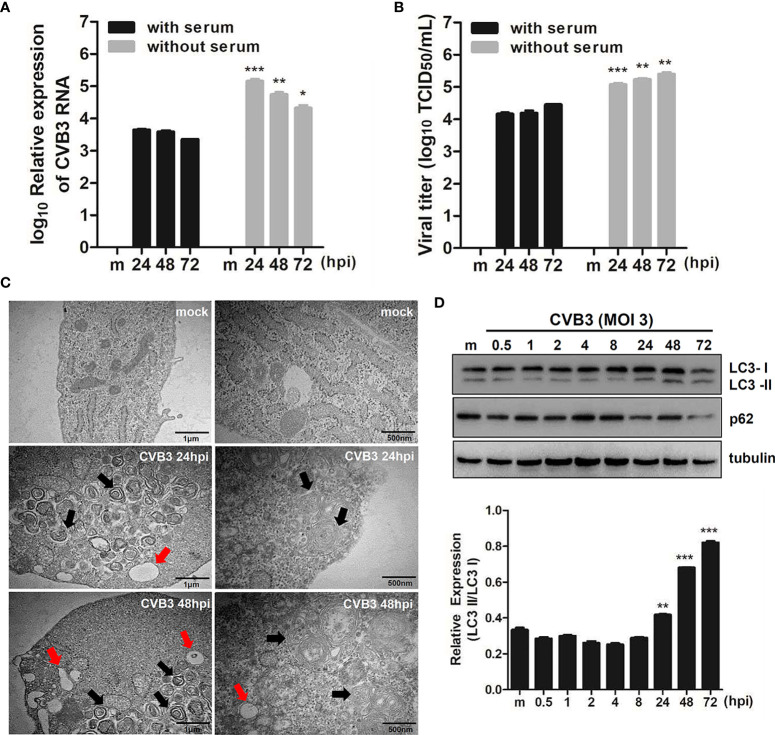
CVB3 infection in hNPCs results in the induction of autophagy. **(A)** hNPCs (with or without serum starvation) were infected with mock or CVB3 at an MOI of 5 for the indicated time points. Quantitative realtime PCR was performed to measure CVB3 RNA copy number. Data are representative of three independent experiments (mean ± SD). **(B)** Supernatant was collected to determine viral titers measured by TCID50 assay. Results represent mean ± SD of two independent experiments. *p < 0.05; **p < 0.01; ***p < 0.001 *vs*. CVB3-infected cells cultured with serum. **(C)** The ultrastructure of intracellular organelles in CVB3-infected cells exposed to serum starvation was observed by transmission electron microscopy. Red arrow indicates lysosomes, and black arrow indicates autophagosomes. **(D)** Immunoblot analysis of LC3 and p62 expression in CVB3-infected cells at the indicated time points. Tubulin expression was examined, which was used as a protein loading control. The ratio of LC3-II to LC3-I expression at the indicated time points after CVB3 infection is shown in the graph, and band intensity was quantitated by densitometric analysis using the ImageJ software.

Our previous study shows that CVB3 targets hNPCs (Oh et al., 2020). To examine whether the induction of quiescence in hNPCs would affect viral replication efficiency, hNPCs were cultured with or without serum before and after CVB3 infection. Both quantitative realtime PCR and TCID50 assay showed relatively increased viral RNA from the cells and viral titer from the supernatants, respectively, under serum starvation compared with results under normal conditions (**Figures 1A, B**).

To examine whether CVB3 infection induces autophagy, we performed TEM analysis following CVB3 infection in hNPCs. Similar to previous reports (Wong et al., 2008; Kemball et al., 2010; Tabor-Godwin et al., 2012), TEM images of CVB3-infected cells revealed increased prevalence of autophagosome-like vesicles in the perinuclear region compared to that in mock-infected cells **(** **Figure 1C** **)**. To further confirm the increase in formation of autophagosomes after viral infection, we examined LC3 and p62 expression levels, another hallmark of autophagy, by immunoblot analysis. As shown in **Figure 1D**, CVB3 infection promoted LC3-II conversion, whereas p62 protein level was gradually diminished upon CVB3 infection, confirming CVB3-mediated induction of autophagy in hNPCs.

### CVB3 Infection Alters Mitochondrial Function and Induces Mitophagy

**Figure 2:**
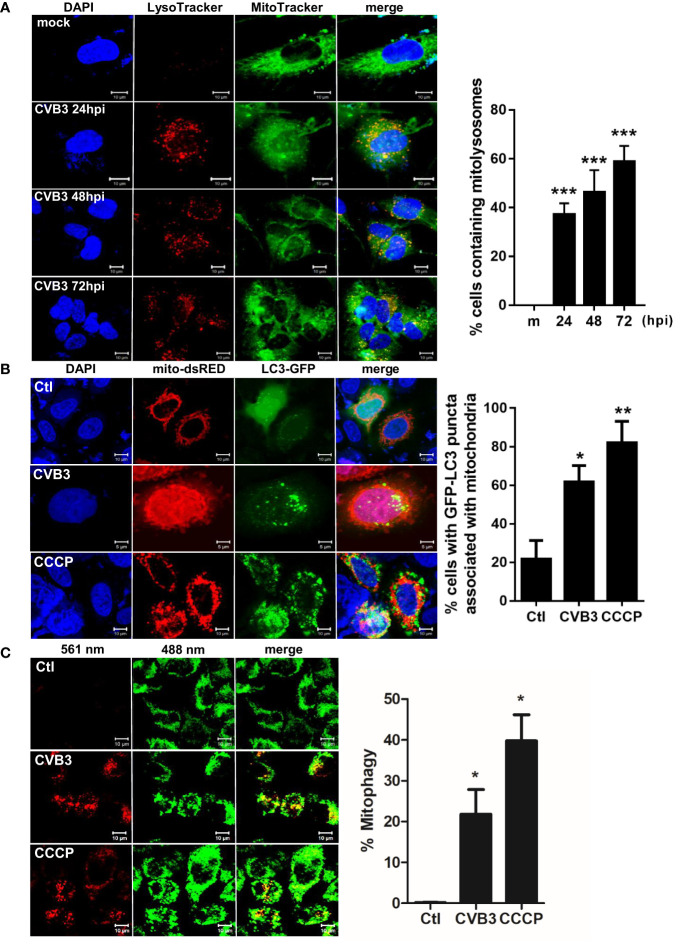
Induction of mitophagy following CVB3 infection. **(A)** hNPCs were infected with mock (m) or CVB3 at an MOI of 5 for the indicated time points. The cells were subsequently stained with MitoTracker Green and LysoTracker Red. Scale bar=10 μm. Percentage (%) of cells containing mitolysosomes is quantified and represented as a graph on the right. At least 100 cells were counted per experiment (n=3) **(B)** HeLa-Parkin cells were transfected with LC3-GFP (green) and mito-dsRED (red) plasmids and infected with CVB3 for 8 h or treated with control DMSO (Ctl) CCCP for 2 h. Scale bar=10 μm. Percentage (%) of cells with LC3 puncta associated with mitochondria is quantified and represented as a graph on the right. **(C)** HeLa-Parkin cells expressing mt-Keima were either treated with 25 μM CCCP for 2 h or infected with CVB3 for 8 h. The emission signal obtained after excitation with the 488 nm laser is shown in green, and that obtained after excitation with the 561 nm laser is shown in red. Zeiss ZEN software was used to determine changes in pH-dependent fluorescence and % mitophagy is represented in graph. Scale bar =10 μm. Bar graph shows mean ± SD from at least 100 cells/condition compiled from three experiments. *p < 0.05; **p < 0.01; ***p < 0.001 *vs*. control DMSO-treated cells.

To characterize the possibility of CVB3-induced mitophagy in cultured hNPCs, we examined the co-localization of mitochondria and lysosomes following CVB3 infection by confocal microscopy analysis. LysoTracker Red and MitoTracker Green are fluorescent probes to specifically detect lysosomes and mitochondria, respectively. We observed co-localization of LysoTracker and MitoTracker signals following CVB3 infection in hNPCs starting at 24 hpi (**Figure 2A**). Given that Parkin-dependent mitophagy depends on the translocation of Parkin to the damaged mitochondria (Narendra and Youle, 2011), we further examined whether CVB3 infection would lead to the translocation of Parkin, an E3 ubiquitin ligase that is a key mediator of mitochondrial quality control processes. Similar to previous findings (Narendra et al., 2010), Parkin was recruited to the mitochondria after CCCP treatment for 2 h, whereas CVB3-infected mitochondria showed Parkin punctate formation in mitochondria and co-localized with LC3-RFP-expressing cells, suggesting that CVB3-induced mitophagy is dependent on Parkin (**Supplementary Figure 1**). Additionally, we examined whether CVB3 infection would result in LC3 punctate formation in mitochondria. We observed significantly increased numbers of LC3-GFP-expressing cells co-immunostained with mito-dsRED-formed punctate structures similar to CCCP-treated cells, suggesting the possibility of mitophagy induction *via* CVB3 infection (**Figure 2B**).

Next, we investigated whether CVB3 infection induces mitophagy, using a pH-sensitive fluorescent protein called mt-Keima. Quantitative measurement of mt-Keima expression was performed with confocal microscopy, allowing us to quantitatively determine lysosomal delivery of mitochondria by measuring the ratio of the area of lysosomal (red) signal to mitochondrial (green) signal. As shown in **Figure 2C**, CVB3 infection led to a significant increase in mitophagy levels, similar to that observed in CCCP-treated cells.

**Figure 3:**
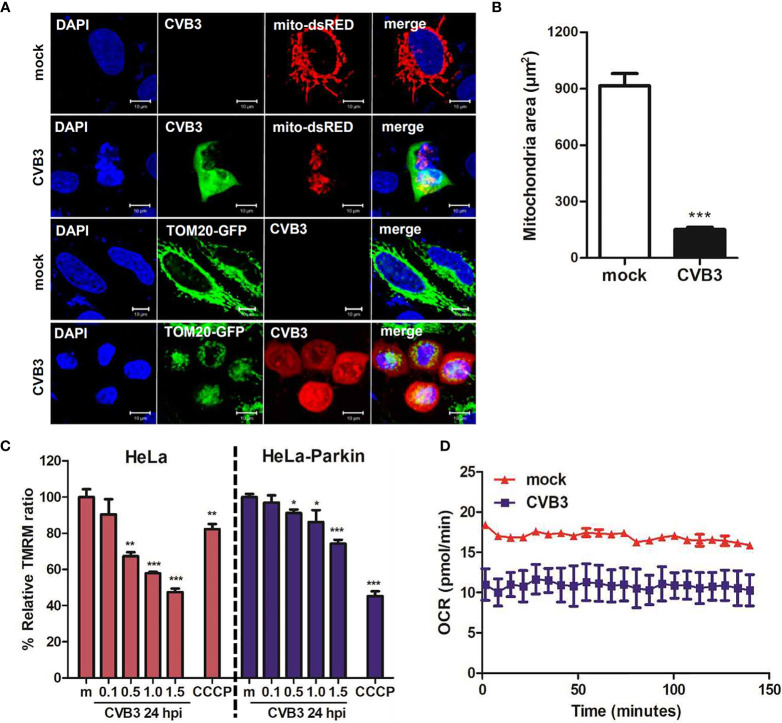
CVB3 infection induces mitochondrial fission and reduces mitochondrial membranes potential. **(A)** HeLa cells were transfected with mitochondrial protein-expressing plasmids (TOM20-GFP or mito-dsRED) and infected with CVB3 for 8 h. CVB3 was immunostained with anti-Coxsackievirus B3 antibody. CVB3-infected cells show fragmented mitochondria. Scale bar = 10 μm. **(B)** Quantification of mitochondrial area as shown in **(A)** is represented (n = 50). **(C)** HeLa or HeLa-Parkin-expressing cells were treated with CCCP for 2 h or infected with mock (m) or CVB3 at different MOIs for 24 h. Carbonyl cyanide m-chlorophenyl hydrazone (CCCP) is a known mitochondrial uncoupler, which reduces mitochondrial membrane potential (MMP). Relative tetramethylrhodamine methyl ester (TMRM) intensity in each group of live cells was measured using a VarioSkan LUX multiplate reader (mean ± SD; n = 3). *p < 0.05; **p < 0.01; ***p < 0.001, *vs*. mock-infected cells. **(D)** Oxygen consumption rate (OCR) in mock or CVB3-infected hNPCs (MOI of 5) was measured in real-time under basal conditions with an XFp Seahorse analyzer. Data represent mean ± SD of three biological replicates.

Given that Sin et al. recently reported that CVB3 associates with mitochondria and concomitantly triggers mitochondrial fragmentation (Sin et al., 2017), we wanted to further investigate whether CVB3 infection would alter mitochondrial dynamics and affect mitochondrial function. Similar to the study by Sin et al., CVB3 infection resulted in increased fragmented mitochondrial networks, leading to augmentation of mitochondrial fission (**Figure 3A**). Quantification analysis of mitochondria indicated a 4-fold decrease in mitochondrial area when cells were infected with CVB3 (**Figure 3B**). Mitophagy is triggered by loss of mitochondrial membrane potential (MMP), and carbonyl cyanide m-chlorophenyl hydrazone (CCCP) is known to induce loss of MMP and generate reactive oxygen species required for the mitophagic process (Villa et al., 2017; Xiao et al., 2017). As demonstrated in **Figure 3C**, CVB3 infection in either HeLa- or HeLa-Parkin cells resulted in a significant loss of MMP, similar to that in CCCP-treated cells. Next, to determine whether the alterations in mitochondrial morphology upon CVB3 infection affect mitochondrial function, we monitored metabolite flux using the Seahorse XFp Analyzer and measured the oxygen consumption rate (OCR), an indicator of cellular respiration, in mock- versus (*vs*) CVB3-infected hNPCs. Compared to mock-infected cells, CVB3-infected cells displayed decreased basal levels of OCR, suggesting that CVB3 may trigger mitochondrial fission and decrease mitochondrial respiration in hNPCs (**Figure 3D**).

**Figure 4:**
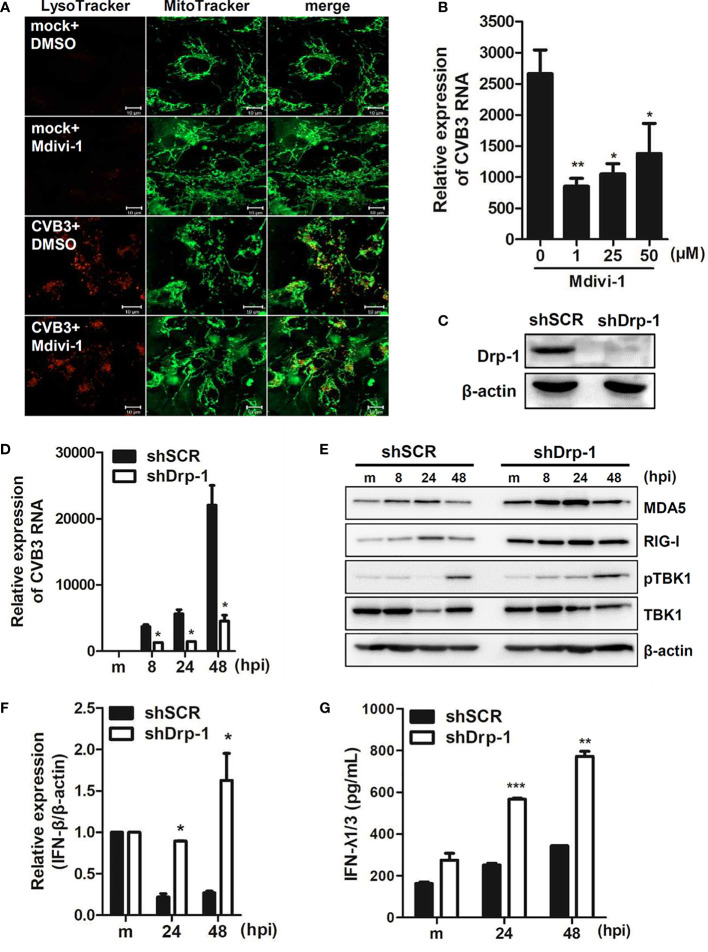
Mitochondrial fission is important for CVB3 replication **(A)** hNPCs were treated with DMSO control or 25 μM Mdivi-1 for 2 h and infected with mock or CVB3 (48 hpi, MOI=5). The cells were subsequently stained with MitoTracker Green and LysoTracker Red. Scale bar = 10 μm. **(B)** hNPCs were pre-treated with different doses of Mdivi-1 for 2 h and infected with CVB3 at an MOI of 5 for 48 h. CVB3 vRNA expression was quantitated, normalized, and analyzed by quantitative realtime PCR (mean ± SD; n = 3). *p < 0.05; **p < 0.01; *vs*. DMSO-treated cells. **(C)** The knockdown efficiency of Drp-1 in scrambled control shRNA (shSCR) and Drp-1-specific shRNA-expressing (shDrp-1) THP-1 cells was confirmed by immunoblot analysis. **(D–G)** THP-1-shSCR and -shDrp-1 cells were infected with mock (m) or CVB3 at an MOI of 5 for the indicated time points. **(D)** CVB3 vRNA expression was quantitated, normalized, and analyzed by quantitative realtime PCR (mean ± SD; n = 3) **(E)**. The protein levels of RIG-I, MDA5, and phospho-TBK/total TBK were measured by immunoblot analysis. **(F)** *IFN-β* mRNA levels were measured by quantitative realtime PCR (mean ± SD; n = 3). **(G)** IFN-λ1/3 secretion levels were measured by ELISA. *p < 0.05; **p < 0.01; ***p < 0.001, compared with THP-1-shSCR cells at the indicated time points.

We further examined whether mitochondrial fission is important for CVB3 replication. Dynamin-like protein 1 (Drp1) is an important mediator of mitochondrial fission. Thus, hNPCs were pre-incubated with Mdivi-1, a Drp-1-mediated fission inhibitor, and infected with CVB3. First, we confirmed that Mdivi-1 treatment led to the inhibition of mitochondrial fission, contributing to elongated mitochondria (**Figure 4A**). Furthermore, Mdivi-1 treatment resulted in diminished vRNA copy number, suggesting the importance of mitochondrial fission for efficient CVB3 replication (**Figure 4B** **).** In addition, to confirm the effect of mitochondrial dynamics on CVB3 replication efficiency, we used human macrophages (THP-1) with stable knockdown of Drp-1 using lentiviral particles expressing scrambled or Drp-1-specific short hairpin RNA (shRNA) (**Figure 4C**). As reported previously (Park et al., 2015), Drp-1-deficient THP-1 cells (shDrp-1) showed an elongated or enlarged mitochondrial morphology as observed by confocal microscopy (data not shown). Interestingly, knockdown of Drp-1 led to the suppression of CVB3 replication compared to scrambled shRNA-expressing (shSCR) THP-1 cells (**Figure 4D** **)**. We next examined whether Drp-1 contributes to host cellular response to CVB3 infection. As shown in **Figure 4E**, Drp-1-deficient cells led to increased protein expression levels of retinoic acid inducible gene-I (RIG-I) and melanoma differentiation antigen 5 (MDA5) compared to shSCR THP-1 cells. Accordingly, the expression of downstream molecules of the RIG-I pathway, such as IFN-β mRNA and IFN-λ1/3 protein, at 24 and 48 hpi (post-infection) was significantly increased, as shown in **Figures 4F, G**. Taken together, these findings indicate the possibility that mitochondrial fission is essential for CVB3 replication, and mitophagy could contribute to controlling viral replication.

**Figure 5:**
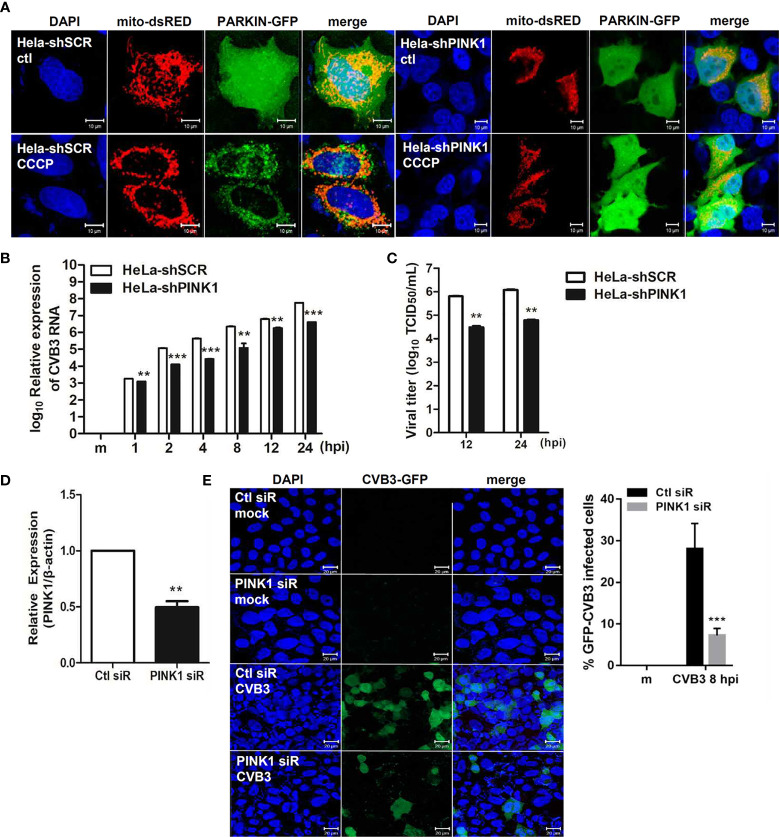
PINK1 is important for CVB3 replication **(A)** HeLa-shSCR and HeLa-shPINK1 cells were treated with CCCP for 2 h. Representative images illustrating PINK1-dependent changes in Parkin-GFP fluorescence from diffuse to punctate form. (Scale bar=10 μm). **(B)** Quantitative realtime PCR results indicating the expression levels of CVB3 vRNA. **p < 0.01; ***p < 0.001 *vs*. HeLa-shSCR-expressing cells. **(C)** Supernatants were collected from HeLa-shSCR and HeLa-shPINK1 cells infected with CVB3 at an MOI of 1 for various time points. Viral titers were determined by TCID50 assay and expressed as TCID50/mL. Results represent mean ± SD of two independent experiments. **(D)** HeLa cells were transfected with control Ctl siR or *PINK1*-specific siRNA PINK1 siR for 24 h and infected with GFP-CVB3 (MOI 1) for 8 h. The effects of siRNA on *PINK1* mRNA levels from each sample is quantified by realtime PCR, and are shown (n=3). **(E)** Percentage of cells expressing GFP-CVB3 was calculated and is represented in the graph. (Scale bar=10 μm) At least 100 cells were counted per experiment (n=3). **p < 0.01; ***p < 0.001, compared with control siRNA-expressing cells.

Given that Parkin translocation to the mitochondria is PINK1-dependent (Vives-Bauza et al., 2010), we next investigated the effect of PINK1 knockdown on CVB3 infection. HeLa and HeLa-shPINK1 cells were treated with CCCP. Knockdown of PINK1 prevented CCCP-induced Parkin translocation to the mitochondria, confirming that Parkin translocation is PINK1-dependent (**Figure 5A**). To address whether PINK1 is essential for CVB3 replication, cells were infected with CVB3 at an MOI of 1 for the indicated timepoints. Quantitative realtime PCR indicated that the deficiency of PINK1 led to a significant reduction in CVB3 vRNA copy numbers (**Figure 5B**). Additionally, CVB3 viral titer was greatly diminished due to the absence of PINK1 expression (**Figure 5C**), suggesting that knockdown of PINK1 reduces CVB3 replication and contributes to inhibition of CVB3-induced mitophagy as well as evasion of innate immune response. In addition, we used PINK1-specific siRNAs to determine the effect of PINK1 knockdown on GFP-CVB3 virus replication. As indicated in **Figure 5D**, PINK1-specific siRNA transfection induced efficient knockdown of PINK1, while there was a significant reduction in GFP-CVB3 expression upon PINK1-specific siRNA treatment compared with control siRNA treatment (**Figure 5E**).

### CCCP-Mediated Mitophagy Activation Facilitates CVB3 Replication *via* Inhibition of IFNs

**Figure 6:**
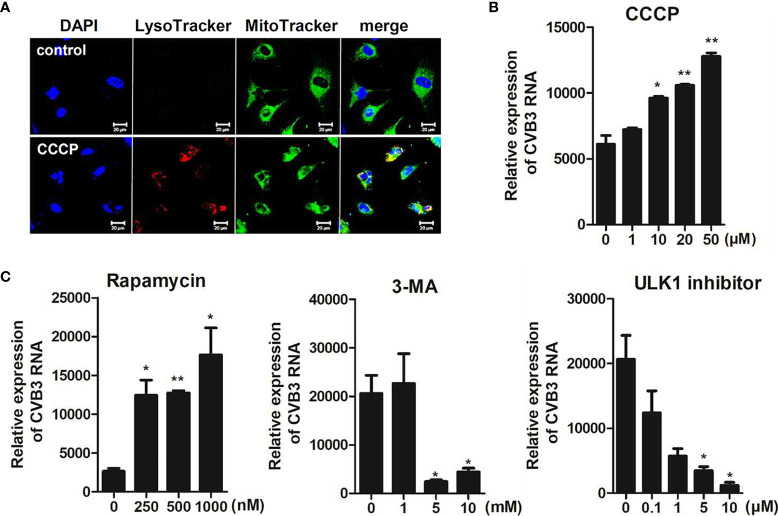
CCCP-mediated activation of mitophagy facilitates CVB3 replication in hNPCs **(A)** hNPCs were treated with 25 μM CCCP for 2 h, and cells were subsequently stained with LysoTracker Red and MitoTracker Green. Scale bar = 20 μm. **(B)** hNPCs were pre-treated with different doses of CCCP for 2 h and infected with CVB3 at an MOI of 5 for 48 h. CVB3 RNA copy number was quantitated, normalized, and analyzed by quantitative realtime PCR (mean ± SD; n = 3). **(C)** hNPCs were treated with rapamycin (autophagy inducer), 3-MA (autophagy inhibitor), or ULK1 inhibitor (SBI-0206965) for 2 h at various concentrations and infected with CVB3 at an MOI of 5 for 48 h. CVB3 RNA copy number was quantitated, normalized, and analyzed by quantitative realtime PCR (mean ± SD; n = 3). *p < 0.05; **p < 0.01, *vs*. DMSO-treated cells.

Next, we determined whether CCCP-mediated activation of mitophagy can affect CVB3 replication and protect against CVB3-mediated cell death. Cells were pre-incubated with different doses of CCCP and infected with CVB3 for 8 h. The protein expression levels of cleaved caspases were detected by immunoblot analysis. The expression levels of cleaved caspase 3 decreased with increasing doses of CCCP (**Supplementary Figure 2**). To confirm the activation of mitophagy following CCCP treatment, hNPCs were stained with LysoTracker Red and MitoTracker Green. Colocalization of mitochondria with lysosomes showed that CCCP treatment successfully induced mitophagy in hNPCs (**Figure 6A**). Interestingly, CCCP treatment also led to the upregulation of CVB3 vRNA expression in a dose-dependent manner (**Figure 6B**). Similarly, cells treated with rapamycin, which has been shown to induce autophagy by inhibiting the mTOR signaling pathway, led to the upregulation of CVB3 replication in a dose-dependent manner, whereas 3-methyladenine (3-MA) or ULK1 inhibitor (SBI-0206965), which are inhibitors of autophagy, led to downregulation of CVB3 replication in hNPCs (**Figure 6C**).

**Figure 7:**
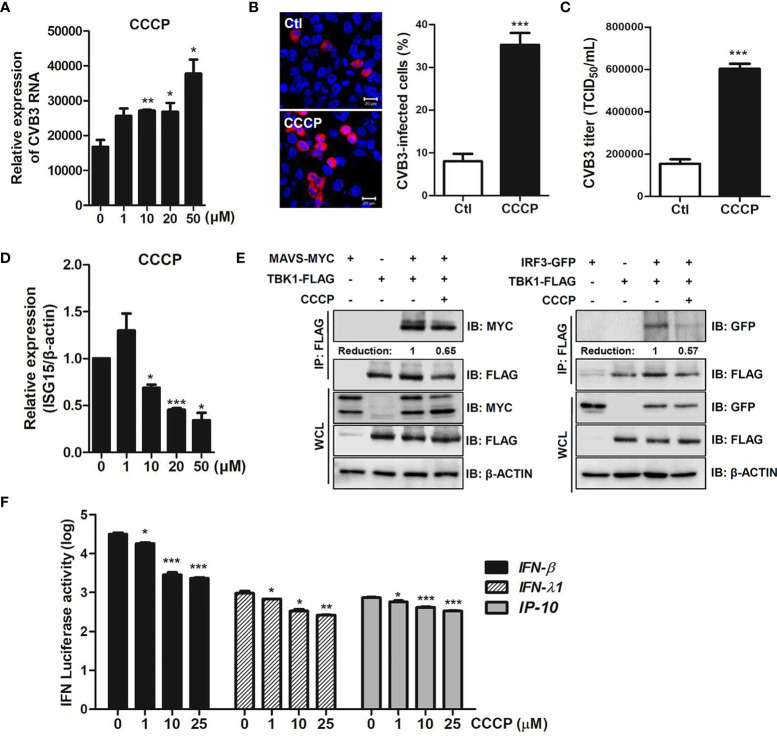
CCCP facilitates CVB3 replication *via* inhibition of host innate immune signaling. **(A)** HeLa-Parkin cells were treated with CCCP at various concentrations and infected with CVB3 at an MOI of 1 for 8 h. CVB3 RNA copy number was quantitated, normalized, and analyzed by quantitative realtime PCR (mean ± SD; n = 3). Statistical analysis: *p < 0.05; **p < 0.01, *vs*. DMSO-treated cells. **(B)** Cells were immunostained with anti-Coxsackievirus B3 antibody and are indicated in red. The images are representative of three independent experiments. CVB3-infected cells were counted and are presented as percentage (%) in the graph. **(C)** Supernatants were collected from HeLa-Parkin cells and viral titers were measured by TCID50 assay. **(D)** HeLa-Parkin cells were treated with CCCP at various concentrations and infected with CVB3 at an MOI of 1 for 8 h. ISG15 mRNA level was measured by quantitative realtime PCR (mean ± SD; n = 3). Statistical analysis: *p < 0.05; ***p < 0.001, *vs*. DMSO-treated cells. **(E)** HeLa-Parkin cells were transfected with MYC-tagged MAVS and TBK1-FLAG (left) or IRF3-GFP and TBK1-FLAG-encoding plasmids and treated with or without 10 μM CCCP for 2h. The media was replaced and cells were incubated for 24 h. Next day, cells were lysed and precipitated using anti-FLAG antibody. The cell lysates and immunoprecipitates were analyzed by immunoblot analysis using anti-FLAG and anti-MYC antibodies. Relative densitometric analysis of immunoblot is presented with normalized densitometric units plotted against the treatment (shown as numbers). WCL, whole cell lysates **(F)** HeLa-Parkin cells were transfected with plasmids encoding RIG-I-FLAG for 24 h and transiently co-transfected with IFN-β, IFN-λ1, and IP-10 luciferase reporter plasmids and a Renilla luciferase reporter plasmid (internal control). After 24 h of transfection, DMSO or CCCP was added to the cells for 2 h. Relative luciferase activity is shown (mean ± SD; n = 3). p < 0.05; **p < 0.01; ***p < 0.001, *vs*. DMSO-treated cells.

We also investigated the role of CCCP-mediated Parkin-dependent mitophagy following CVB3 infection. Quantitative realtime PCR analysis showed a significant increase in the RNA copy number of CVB3 following CCCP treatment in a concentration-dependent manner in HeLa-Parkin cells compared to the DMSO control (**Figure 7A**). Additionally, both confocal microscopy analysis and TCID50 assay indicated a significant increase in CVB3 replication and infectivity following the induction of mitophagy by CCCP treatment (**Figures 7B, C**).

Bu et al. recently reported that Parkin is a negative regulator of antiviral immunity *via* RIG-I/MDA5 degradation (Bu et al., 2020). Given that CCCP-mediated mitophagy influences CVB3 replication efficiency, we sought to determine possible mechanisms by which CCCP facilitates viral replication. Interestingly, mRNA expression of antiviral genes, such as *interferon stimulated gene 15 (ISG15)*, was robustly suppressed upon CCCP treatment of CVB3-infected HeLa-Parkin cells **(** **Figure 7D**). The effect of CCCP on the RIG-I like receptor (RLR) pathways was evaluated by co-immunoprecipitation assay. HeLa-Parkin cells were transiently transfected with the following plasmids; MAVS and TBK1 or IRF3 and TBK1-encoding plasmids and treated with CCCP for 2 h. As shown in **Figure 7E**, the interaction between MAVS and TBK1 or IRF3 and TBK1 was greatly attenuated by CCCP treatment, suggesting that CCCP-mediated mitophagy leads to diminished RLR signaling interactions. Moreover, the luciferase reporter assay indicated that RLR-triggered IFN-β, IFN-λ1, and IP-10 promoter activities were significantly attenuated upon CCCP treatment in HeLa-Parkin cells (**Figure 7F**).

**Figure 8:**
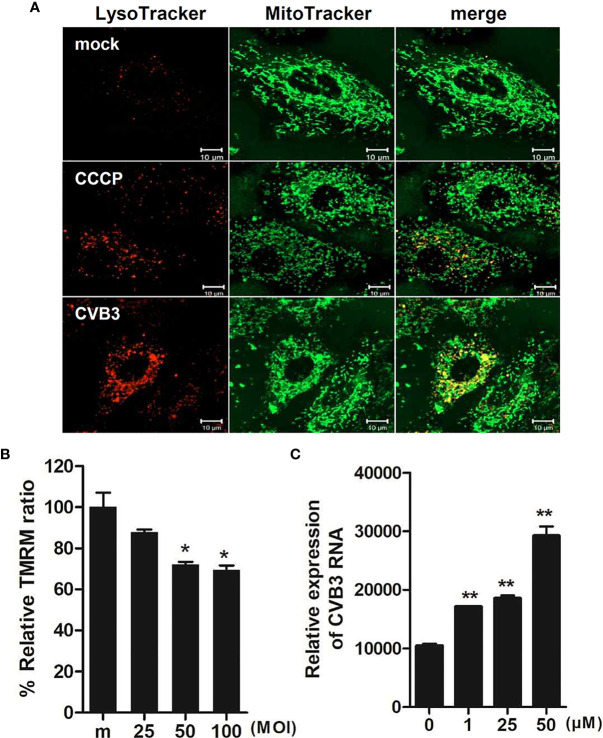
Effect of CCCP-mediated mitophagy following CVB3 infection in H9C2 cardiomyocytes **(A)** H9C2 cells were treated with 25 μM CCCP for 2 hours or infected with mock or CVB3 for 24 h (MOI =50). The cells were subsequently stained with MitoTracker Green and LysoTracker Red. Scale bar=10 μm. **(B)** H9C2 cells were infected with CVB3 at different MOIs for 24 h. Relative tetramethylrhodamine methyl ester (TMRM) intensity in each group of live cells was measured using a VarioSkan LUX multiplate reader (mean ± SD; n = 2). *p < 0.05, *vs*. mock-infected cells **(C)** H9C2 cells were treated with CCCP at various concentrations and infected with CVB3 at an MOI of 50 for 24 h. CVB3 RNA copy number was quantitated, normalized, and analyzed by quantitative realtime PCR (mean ± SD; n = 2). Statistical analysis: *p < 0.05; **p < 0.01, *vs*. DMSO-treated cells.

Given that CVB3 is thought to be the most commonly identified virus that causes viral myocarditis (Blyszczuk, 2019), we next examined whether CVB3 infection would trigger mitophagy in cardiomyocytes. H9C2 cardiomyocytes were treated with CCCP or infected with CVB3 (MOI =50). Confocal microscopy analysis show that CVB3-infected cardiomyocytes displayed colocalization of mitochondria with lysosomes, confirming the formation of mitolysosomes (**Figure 8A**). We also investigated whether CVB3 infection would cause MMP changes in H9C2 cardiomyocytes. A significant loss of MMP was detected with increasing MOIs of CVB3, indicating that CVB3 infection causes mitochondrial membrane depolarization in cardiomyocytes (**Figure 8B**). To verify whether CCCP treatment can affect CVB3 replication, the expression of CVB3 RNA was quantified by quantitative realtime PCR (**Figure 8C**). Elevated CVB3 replication was detected with higher CCCP concentrations, suggesting that CCCP-mediated mitophagy significantly facilitates CVB3 replication in cardiomyocytes.

## Discussion

Although mounting evidence suggests that defective mitophagy may contribute to development of aging, cancer and neurological disorders, the effect of mitophagy on viral infection are just beginning to be examined. Recent studies suggest that multiple viruses develop a wide range of strategies to subvert and benefit from mitophagy (Kim et al., 2013a; Kim et al., 2013b; Xia et al., 2014; Li et al., 2016; Ding et al., 2017; Gou et al., 2017; Sin et al., 2017; Zhang et al., 2018; Vilmen et al., 2020; Wang et al., 2020). The first evidence of virus-triggered mitophagy was presented in hepatitis B and C viral infection models. Kim et al. reported that both hepatitis B and C viruses can induce mitochondrial fission and inhibit apoptosis to promote viral survival in a Parkin-dependent manner (Kim et al., 2013a; Kim et al., 2013b; Kim et al., 2014). Similarly, mitophagy was triggered to promote viral persistence by viruses such as porcine reproductive and respiratory syndrome virus, classical swine fever virus, and CVB3 (Li et al., 2016; Gou et al., 2017; Sin et al., 2017). In particular, Sin et al. reported that CVB3 localizes to mitochondria and triggers mitophagy and this mitophagy-driven extracellular vesicles can act as a viral dissemination machinery to prolong viral replication.

The role of autophagy during CVB3 infection has been previously examined by several groups (Wong et al., 2008; Tabor-Godwin et al., 2012; Robinson et al., 2014). CVB3 was demonstrated to escape from the host cells *via* extracellular vesicles, given the observation that extracellular vesicles containing infectious virus particles are released from various progenitor cell types by inducing autophagy (Robinson et al., 2014). Meanwhile, Tabor-Godwin et al. showed that CVB3 infection in HL-1 cardiomyocytes resulted in an increase in autophagic signaling, and viral titers increased upon rapamycin treatment. On the contrary, no change occurred in autophagic signaling following CVB3 infection in mouse undifferentiated NPCs. However, in differentiated NPCs, a decrease in autophagy signaling was observed following CVB3 infection (Tabor-Godwin et al., 2012). Interestingly, in our study, rapamycin treatment led to increased CVB3 replication in undifferentiated hNPCs, and we demonstrated the ability of CVB3 to effectively replicate in hNPCs and induce mitophagy to facilitate their replication. This discrepancy may be due to the fact that Tabor-Godwin et al. used NPCs isolated from mice, whereas we used iPSC-driven human NPCs. Hence, the role of autophagy in modulating viral replication may differ between human and mouse-derived cells. Furthermore, different sources of the CVB3 strain may have resulted in different outcomes regarding the role of autophagy.

We recently showed that hNPCs are susceptible to CVB3 infection, and CVB3 infection in these cells results in the activation of RLR signaling pathways (Oh et al., 2020). Our current study further expands our knowledge of how CVB3 may regulate innate immune response *via* mitophagy induction, using several in-depth quantitative analysis to measure changes in mitochondrial dynamics. In lines with several earlier reports, we first observed the formation of autophagosomes following CVB3 infection in hNPCs. Moreover, a marked punctate LC3 distribution was localized within the mitochondria, which increased in a time-dependent manner. Given that CVB3 infections are closely linked to myocarditis, which can lead to excessive myocardial inflammation, the role of mitophagy in cardiomyocytes were also investigated in our study. Similar to results from hNPCs or HeLa cells, altered mitochondrial dynamics associated with infection CVB3 resulted in depolarization of MMP and mitophagy promotion in CVB3-infected H9C2 cardiomyocytes. Although recent progress in molecular mechanisms of mitophagy has greatly improved our understanding of mitochondrial quality control in the heart (Fan et al., 2020), a potential role of mitophagy contributing to the pathogenesis of viral myocarditis needs further investigation.

Mitophagy selectively eliminates impaired mitochondria and targets them to the lysosomes for degradation (Wong et al., 2019). PINK1/Parkin-mediated pathways are widely accepted as the main mechanisms that regulate mitophagy (McWilliams and Muqit, 2017). Following the depolarization of MMP (Δψm), PINK1 is stabilized and recruits Parkin to impaired mitochondria. Parkin then leads to ubiquitination of multiple substrates and triggers the removal of damaged mitochondria. Several viruses have been known to trigger mitophagy directly or indirectly and control mitophagic processes to promote persistent infection and suppress innate immune responses (Zhang et al., 2018). As reported by Sin et al. (2017), we also observed Drp-1-mediated fragmentation of mitochondria following CVB3 infection. CVB3 infection also induced Parkin accumulation in the mitochondria and increased the number of cells with Parkin translocated to the mitochondria. These data support that CVB3 localizes to mitochondria, induces PINK1/Parkin-mediated mitophagy, and subsequently egress from the host cell. Furthermore, we showed that mitophagy induction dampens the type I and III IFN signaling pathways by affecting the interaction between MAVS and TBK1 as well as TBK1 and IRF3. These results are in line with a recent finding from Bu et al. (2020), which suggested that CCCP-mediated mitophagy negatively regulates antiviral immunity *via* activation of Parkin-mediated K48-linked polyubiquitination, facilitating the degradation of RIG-I and MDA5. Given that aggregated MAVS acts as a recruitment platform that activates downstream signaling molecules such as TBK1, it is plausible that CVB3 may facilitate mitophagy to selectively inhibit the mitochondrial or peroxisomal localization of antiviral molecules, such as MAVS and degrade antiviral platforms like mitochondria or peroxisomes. Furthermore, given that multiple mitophagy receptors like BNIP3, NIX, and FUNDC1 promote the selective elimination of damaged mitochondria in various pathological conditions, it will also be interesting to determine if CVB3 infection can trigger these mitophagy receptors and whether these receptor functions are ubiquitous or limited to specific tissues and cell types.

Considering that mitophagy contributes to viral replication by antagonizing IFN response, additional *in vitro* and *in vivo* studies to further investigate the role of mitophagy during the progression of CVB3 infection and persistence in the heart and central nervous system may be critical to provide clues for new therapeutic treatments. Our findings expand the current understanding of the role of mitophagy, facilitating virus infection, which could provide new therapeutic advances to combat CVB3 infection.

## Data Availability Statement

The original contributions presented in the study are included in the article/**Supplementary Material**. Further inquiries can be directed to the corresponding authors.

## Author Contributions

Conceptualization, OS. Methodology, S-JO and JY. Formal analysis, S-JO. Investigation, S-JO. resources, JY and B-KL. Writing—original draft preparation, OS. Writing—review and editing, JY and OS. Visualization, S-JO and OS. Supervision, OS. Project administration, OS. Funding acquisition, OS. All authors contributed to the article and approved the submitted version.

## Funding

This research was funded by the Basic Science Research Program of the National Research Foun-dation of Korea (NRF) by the Ministry of Science, ICT & Future Planning (NRF-2019R1A2C1005961) (to OS) and the Korea government (MSIT) (No. 2016R1A5A2007009) (to JY). Korea Health Technology R&D Project through the Korea Health Industry Development Institute (KHIDI), funded by the Ministry of Health & Welfare, Republic of Korea, (HI21C1252) to S-JO.

## Conflict of Interest

The authors declare that the research was conducted in the absence of any commercial or financial relationships that could be construed as a potential conflict of interest.
