# Corrigendum: CVB3-Mediated Mitophagy Plays an Important Role in Viral Replication *via* Abrogation of Interferon Pathways

**DOI:** 10.3389/fcimb.2021.757341

**Published:** 2021-09-10

**Authors:** Soo-Jin Oh, Byung-Kwan Lim, Jeanho Yun, Ok Sarah Shin

**Affiliations:** ^1^BK21 Graduate Program, Department of Biomedical Sciences, College of Medicine, Korea University Guro Hospital, Seoul, South Korea; ^2^Department of Biomedical Science, Jungwon University, Goesan-gun, South Korea; ^3^Department of Translational Biomedical Sciences, Peripheral Neuropathy Research Center, College of Medicine, Dong-A University, Busan, South Korea

**Keywords:** Coxsackievirus B3 virus (CVB3), mitochondrial dynamics, mitophagy, interferon, neural progenitor cells

## Missing Funding

In the original article, we neglected to include the funder ** Korea Health Technology R&D Project through the Korea Health Industry Development Institute (KHIDI), funded by the Ministry of Health & Welfare, Republic of Korea **, **(HI21C1252)** to **SJO**.

The authors apologize for this error and state that this does not change the scientific conclusions of the article in any way. The original article has been updated.

## Error in Figure/Table

In the original article, there was a mistake in **Figure 2** as published. ** The fluorescence laser of **Figure 2C** were mistakenly mislabeled. The red signal should be labeled as 561 nm and the green signal should be labeled as 488nm**. The corrected **Figure 2** appears below.

**Figure 2:**
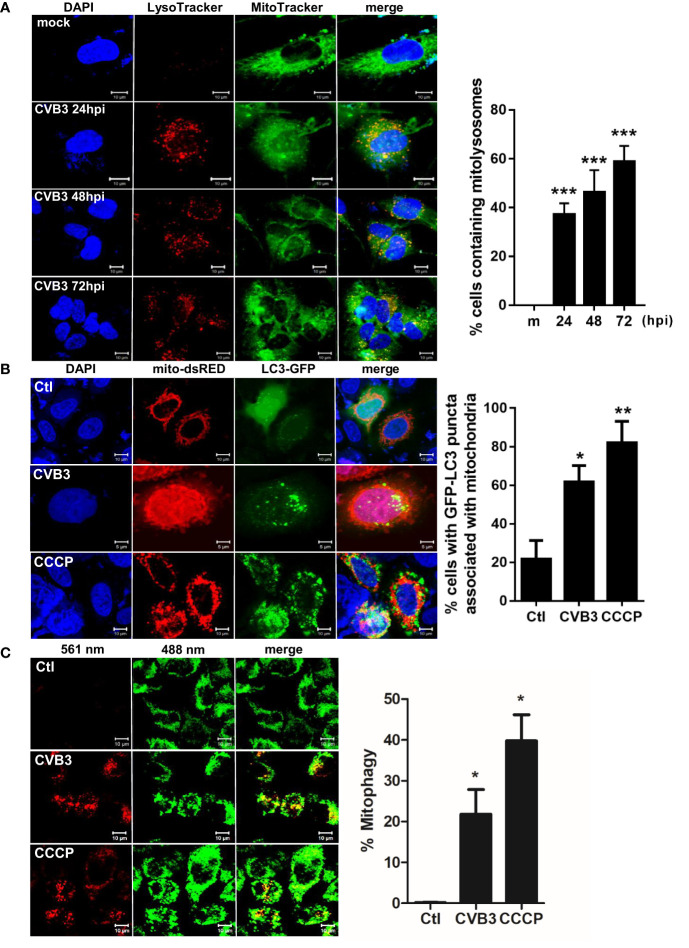
Induction of mitophagy following CVB3 infection. **(A)** hNPCs were infected with mock (m) or CVB3 at an MOI of 5 for the indicated time points. The cells were subsequently stained with MitoTracker Green and LysoTracker Red. Scale bar=10 μm. Percentage (%) of cells containing mitolysosomes is quantified and represented as a graph on the right. At least 100 cells were counted per experiment (n=3) **(B)** HeLa-Parkin cells were transfected with LC3-GFP (green) and mito-dsRED (red) plasmids and infected with CVB3 for 8 h or treated with control DMSO (Ctl) CCCP for 2 h. Scale bar=10 μm. Percentage (%) of cells with LC3 puncta associated with mitochondria is quantified and represented as a graph on the right. **(C)** HeLa-Parkin cells expressing mt-Keima were either treated with 25 μM CCCP for 2 h or infected with CVB3 for 8 h. The emission signal obtained after excitation with the 488 nm laser is shown in green, and that obtained after excitation with the 561 nm laser is shown in red. Zeiss ZEN software was used to determine changes in pH-dependent fluorescence and % mitophagy is represented in graph. Scale bar =10 μm. Bar graph shows mean ± SD from at least 100 cells/condition compiled from three experiments. *p < 0.05; **p < 0.01; ***p < 0.001 *vs*. control DMSO-treated cells.

The authors apologize for this error and state that this does not change the scientific conclusions of the article in any way. The original article has been updated.

Figures, tables, and images will be published under a Creative Commons CC-BY licence and permission must be obtained for use of copyrighted material from other sources (including re-published/adapted/modified/partial figures and images from the internet). It is the responsibility of the authors to acquire the licenses, to follow any citation instructions requested by third-party rights holders, and cover any supplementary charges.

## Publisher’s Note

All claims expressed in this article are solely those of the authors and do not necessarily represent those of their affiliated organizations, or those of the publisher, the editors and the reviewers. Any product that may be evaluated in this article, or claim that may be made by its manufacturer, is not guaranteed or endorsed by the publisher.

