# Supplementary material for: CVB3-Mediated Mitophagy Plays an Important Role in Viral Replication via Abrogation of Interferon Pathways
Source: Front Cell Infect Microbiol. 2021 Jul 6;11:704494. doi: 10.3389/fcimb.2021.704494 (PMC8292102; doi:10.3389/fcimb.2021.704494)
Supplement: Supplementary file 1 [file Table_1.docx]

Supplementary Material

# Supplementary Data

# Supplementary Figures and Tables

## Supplementary Figures

**Supplementary Figure 1. CVB3-induced mitophagosome formation (A)** HeLa cells were transfected with Parkin-GFP and mito-dsRED plasmids and infected with CVB3 for 8 h or treated with CCCP for 2 h. Scale bar = 10 μm. (B) HeLa cells were transfected with Parkin-GFP and LC3-RFP plasmids and infected with mock (m) or CVB3 for 8 h or treated with CCCP for 2 h. Scale bar = 10 μm.


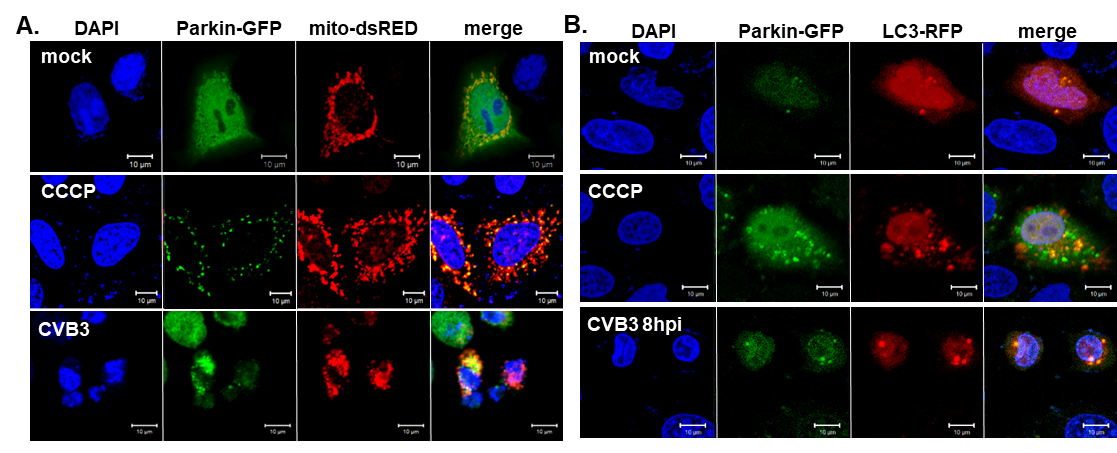


**Supplementary Figure 2. CCCP-mediated activation of mitophagy reduces apoptosis** (A) hNPCs were treated with CCCP for 2 h and infected with CVB3 for 8 h. Cell lysates were analyzed by western blotting for cleavage of caspases. Data representative of three independent experiments are shown.

**
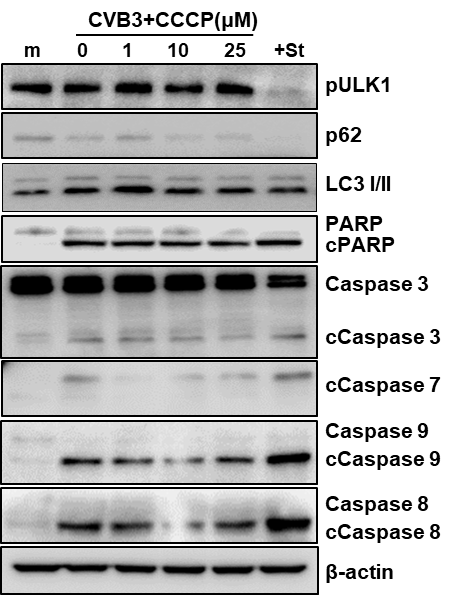
**
